# Long-Term Psychosocial Consequences of Whole-Body Magnetic Resonance Imaging and Reporting of Incidental Findings in a Population-Based Cohort Study

**DOI:** 10.3390/diagnostics12102356

**Published:** 2022-09-28

**Authors:** Dorina Korbmacher-Böttcher, Fabian Bamberg, Annette Peters, Birgit Linkohr, Karl-Heinz Ladwig, Lars Schwettmann, Sabine Weckbach, Christopher L. Schlett, Susanne Rospleszcz

**Affiliations:** 1 Department of Diagnostic and Interventional Radiology, Medical Center-University of Freiburg, Faculty of Medicine, University of Freiburg, 79106 Freiburg, Germany; 2 Institute of Epidemiology, Helmholtz Zentrum München-German Research Center for Environmental Health (GmbH), 85764 Neuherberg, Germany; 3 Institute of Medical Information Sciences, Biometry and Epidemiology(IBE), Ludwig-Maximilians University, 80539 Munich, Germany; 4 German Centre for Cardiovascular Research (DZHK), Partner Site Munich Heart Alliance, 80802 Munich, Germany; 5 Department of Psychosomatic Medicine and Psychotherapy, Klinikum Rechts der Isar, Technische Universität München (TUM), 81675 Munich, Germany; 6 Institute of Health Economics and Health Care Management, Helmholtz Zentrum München, German Research Center for Environmental Health, 85764 Neuherberg, Germany; 7 Department of Economics, Martin Luther University Halle-Wittenberg, 06099 Halle (Saale), Germany; 8 Department of Diagnostic and Interventional Radiology, University Hospital Heidelberg, 69126 Heidelberg, Germany

**Keywords:** mental health, incidental findings, magnetic resonance imaging, whole body imaging, longitudinal study

## Abstract

Management of radiological incidental findings (IF) is of rising importance; however, psychosocial implications of IF reporting remain unclear. We compared long-term psychosocial effects between individuals who underwent whole-body magnetic resonance imaging (MRI) with and without reported IF, and individuals who did not undergo imaging. We used a longitudinal population-based cohort from Western Europe. Longitudinal analysis included three examinations (exam 1, 6 years prior to MRI; exam 2, MRI; exam 3, 4 years after MRI). Psychosocial outcomes included PHQ-9 (Patient Health Questionnaire), DEEX (Depression and Exhaustion Scale), PSS-10 (Perceived Stress Scale) and a Somatization Scale. Univariate analyses and adjusted linear mixed models were calculated. Among 855 included individuals, 25% (n = 212) underwent MRI and 6% (n = 50) had at least one reported IF. Compared to MRI participants, non-participants had a higher psychosocial burden indicated by PHQ-9 in exam 1 (3.3 ± 3.3 vs. 2.5 ± 2.3) and DEEX (8.6 ± 4.7 vs. 7.7 ± 4.4), Somatization Scale (5.9 ± 4.3 vs. 4.8 ± 3.8) and PSS-10 (14.7 ± 5.7 vs. 13.7 ± 5.3, all *p* < 0.05) in exam 3. MRI participation without IF reporting was significantly associated with lower values of DEEX, PHQ-9 and Somatization Scale. There were no significant differences at the three timepoints between MRI participants with and without IF. In conclusion, individuals who voluntarily participated in whole-body MRI had less psychosocial burden and imaging and IF reporting were not associated with adverse long-term psychosocial consequences. However, due to the study design we cannot conclude that the MRI exam itself represented a beneficial intervention causing improvement in mental health scores.

## 1. Introduction

Whole-body magnetic resonance imaging (MRI) is increasingly utilized not only in population-based research [1,2,3], but also in clinical care such as screenings of oncological patients with prostate cancer or multiple myeloma [4,5,6], for instance, as well as in direct-to-consumer preventive health screenings [7,8,9,10]. Advantages of MRI in these settings include the lack of ionizing radiation, the high anatomic coverage, and technical developments providing decreased examination times and a higher soft-tissue resolution [11]. Whole-body MRI leads to a substantial number of incidental findings (IF), which are unexpected discoveries unrelated to the objectives of the examination but with potential health consequences for the study participant [12]. The range of detected IF varies dependent on the imaging protocol and type of cohort. In population-based whole-body MRI, IF were found in up to 35% of participants [9,13].

However, especially in population-based imaging research, a substantial number of IF turn out to be without clinical consequences or even false positive due to the lower pretest probability of pathological findings in contrast to patient cohorts [13,14,15,16]. For instance, only about 1/5 of potentially serious incidental findings were associated with serious final diagnoses in the population-based UK Biobank Imaging Study [17]. This is especially problematic as waiting for or receiving an IF report can cause substantial distress for participants [18,19,20]. Furthermore, disclosure of IF may have medical, financial and psychosocial consequences [15,16].

Nevertheless, research participants mainly wish for disclosure of IF, as gaining information about their own health is frequently the main motivation for participation in a research study besides the contribution to a scientific purpose [18,19,20,21]. Disclosing IF has the potential to provide information about previously unknown serious diagnoses such as malignancies to participants and clinical caregivers, which might improve the course of the disease [8,17].

Consequently, research investigators and clinical caregivers are confronted with ethical problems when addressing a balance between overreporting of IF and withholding information from participants [21,22,23,24]. Furthermore, a high number of reported IF might also have a crucial financial impact on the health care system due to further examinations such as biopsies and follow-up imaging [15,16,25].

In a previous study based upon a whole-body MRI-study within the population-based Cooperative Health Research in the Region of Augsburg (KORA), adverse short-term psychosocial consequences of IF disclosures showed to be limited as there was no significant increase in depression assessed by PHQ-9 (Patient Health Questionnaire) six months after the MRI examination [18]. Studies addressing the psychosocial impact of IF, however, are rare, especially with respect to long-term implications. Thus, we now aimed to analyze long-term psychosocial effects of MRI participation and IF disclosure within a population-based cohort.

## 2. Materials and Methods

### 2.1. Study Design, Sample and Endpoint Assessment

The KORA-MRI sample comprises 400 participants, who underwent whole-body MRI during the second follow-up examination (KORA-FF4, N = 2278) of the original KORA-S4 study in 2013/2014 (exam 2) [1]. The KORA-MRI study was designed to investigate subclinical disease burden in individuals with prediabetes and diabetes [1]. Exclusion criteria for MRI participation were age > 73 years, prior cardiovascular disease, or any contraindications to the MRI procedure, such as metal parts inside the body, claustrophobia, or allergy to the contrast agent [1]. Imaging revealed 113 clinically relevant incidental findings (IF) in 89 participants by reading of board-certified radiologists with unclear liver lesion, silent myocardial infarction and complex renal cyst being the most frequently reported IF [18]. The standardized IF reporting process has been described previously [18].

**Figure 1:**
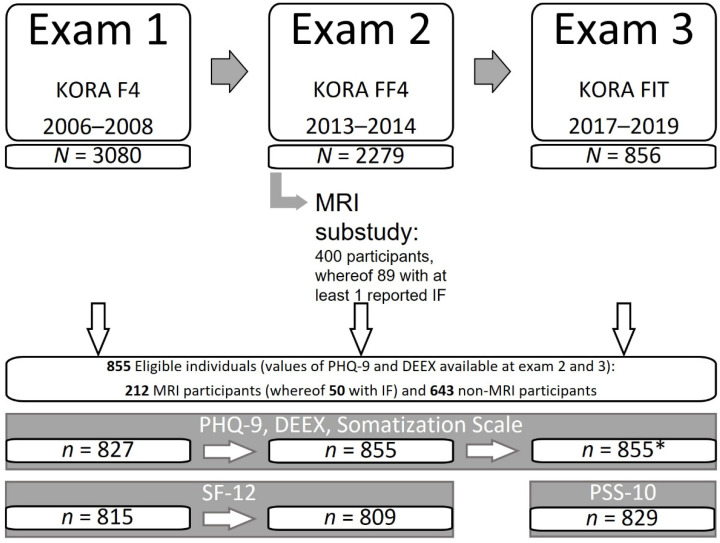
Schematic Study Design, Sample and Endpoint Assessment. Of 3080 individuals from exam 1, only 856 participated in exam 3. Of these, 855 could be included in the main analysis. The Patient Health Questionnaire (PHQ-9), the Depression and Exhaustion Scale (DEEX) and the Somatization Scale were available at all three exams. The Short-Form-Health-Survey-12 (SF-12) was applied at exams 1 and 2, and the Perceived Stress Scale (PSS-10) at exam 3. * denotes n = 854 for Somatization Scale at exam 3.

Examinations of the KORA-FF4 study sample (i.e., of both the KORA-MRI participants and those who did not participate in KORA-MRI) were also performed six years prior to MRI (KORA F4, exam 1) and four years after MRI (KORA FIT, exam 3). Study design, sampling methods and data compilation are specified in previous publications [1,18,26,27]. Our analysis thus includes three time points. For the analysis sample, we included all individuals who participated in all three exams and had values of PHQ-9 and DEEX (Depression and Exhaustion Scale) available at least at exam 2 and 3 (compare Figure 1). We denote those individuals who did not undergo the MRI examination as “control group”.

All KORA studies are approved by the ethics committee of the Bavarian Medical Association in Munich, Germany. The KORA-MRI sub-study was additionally approved by the institutional review board of the medical faculty of Ludwig-Maximilians-University Munich, Germany. Investigations were carried out in accordance with the Declaration of Helsinki. Written informed consent was obtained from all individuals in this study.

All interviews, questionnaires and physical examinations were conducted according to highly standardized protocols by trained stuff and underwent rigorous quality control in line with the German guidelines for Good Scientific Practice [28]. 

Psychosocial and health assessments included the following standardized self-rated questionnaires: The Depression and Exhaustion Scale (DEEX) comprises eight items representing nervousness and anxiety, tiredness, fatigue, and lack of concentration with a scoring range of 0 to 24 [29]. The Somatization Scale was obtained as a continuous score with a range from 0–27 and a response scale from 0 (“not at all“) to 3 (“severe“) based on nine symptoms of somatization such as tachycardia and heavy sweating [30,31]. We also assessed depression by applying the Patient Health Questionnaire (PHQ-9), a nine-symptom checklist scoring each depressive symptom from 0 (“not at all“) to 3 (“nearly every day“) leading to a range of 0–27 [32,33]. The Perceived Stress Scale (PSS-10) measures to which extent life has been experienced as uncontrollable during the past month using a response scale ranging from 0 (“never”) to 4 (”very often”) with higher overall scores representing higher levels of perceived stress [34,35]. To investigate quality of life, we used the Short-Form-Health-Survey-12 (SF-12), which assesses the participant’s perception of physical ability, bodily pain, and vitality as well as emotional, social, and mental health creating two subscales, the physical component summary score (PCS) and the mental component summary score (MCS), with higher scores indicating a better quality of life [36,37]. 

DEEX, PHQ-9 and Somatization Scale were assessed at all three exams by the same questionnaires. SF-12 was only assessed at exam 1 and exam 2, again by the same questionnaires, and PSS-10 was merely assessed at exam 3. As the main focus of the present analysis was the psychosocial development after the MRI exam, exclusion criteria were missing values of PHQ-9 and/or DEEX at exam 2 or 3.

Furthermore, at all three exams, we investigated participants’ demographic characteristics such as age, sex, and body mass index (BMI). Family status, smoking status and physician-diagnosed diabetes mellitus were assessed by self-report. Physical activity was defined as >1h of self-reported regular physical activity per week. Antidepressant medication included Anatomical Therapeutic Chemical (ATC) codes N06A [A, B, F, G, X]. Hypertension was defined as blood pressure ≥ 140/90 or intake of antihypertensive medication.

### 2.2. Statistical Analysis

Demographics and psychosocial outcomes are reported as arithmetic mean and standard deviation for continuous variables, and as counts and percentages for categorical variables. Differences between MRI participants and the control group and between MRI participants with and without IF were analyzed by unpaired t-test, Mann–Whitney U test, or χ^2^-test, where appropriate. Furthermore, we compared demographics of included and excluded individuals. *p*-values for longitudinal trends of outcomes in PHQ-9, DEEX and the Somatization Scale were generated using repeated measures analysis of variance (ANOVA) for MRI participants, participants with/without IF, and the control group. To assess the association of MRI participation and IF reporting with trajectories of psychosocial outcomes, linear mixed models with random intercept per individual were calculated and adjusted for age, sex, BMI, hypertension, smoking status, diabetes mellitus, intake of antidepressants, physical activity, and family status. Choice of adjustment variables was guided by prior knowledge; no selection by pre-testing on the current data was done. A *p*-value < 0.05 indicated statistical significance. SPSS Version 26 and R Version 4.0.5 were used for analyses.

## 3. Results

### 3.1. Study Population and Demographic Characteristics

Our final analytical sample contained 212 MRI participants of whom 50 had at least one reported IF. The control group comprised 643 individuals who did not participate in the MRI examination.

Exclusion of individuals was mainly due to missing data in exam 3. In exam 2, excluded MRI participants were on average 4.8 years younger than included subjects (53.4 ± 11.5 years, n = 188, and 58.2 ± 5.6 years, n = 212, respectively) (*p* < 0.001) and were less commonly hypertensive compared to included MRI participants (28%, n = 52, vs. 40%, n = 84) (*p* = 0.02). Individuals who were excluded from the control group were on average 4.9 years older than included individuals in exam 2 (62.9 ± 14.9 years, n = 1236, and 58 ± 5.5, n = 643, respectively) (*p* < 0.001), less commonly stated physical activity (53%, n = 653, vs. 64%, n = 414) (*p* < 0.001) and were more frequently suffering from diabetes mellitus (18%, n = 222, vs. 8%, n = 52) (*p* < 0.001) and hypertension (44%, n = 545, vs. 33%, n = 211) (*p* < 0.001). Furthermore, the percentage of men in exam 2 was higher in the group of excluded participants than in the control group (49%, n = 600, vs. 42%, n = 271) (*p*=0.01). Further demographic characteristics of excluded participants are shown in Appendix A Table A1.

In exam 2, the mean age of MRI participants was 58.2 ± 5.6 years and the mean BMI was 28.2 ± 4.7 kg/m^2^, while the mean age of individuals in the control group was 58 ± 5.5 years and the mean BMI was 27.6 ± 5.3 kg/m^2^. The percentage of individuals with diabetes mellitus in exam 2 was higher in MRI participants (14%, n = 29) than in the control group (8%, n = 52), as well as the percentage of men (55%, n = 117, vs. 42%, n = 271).

The mean age of MRI participants with reported IF in exam 2 was 59.6 ± 5 years, while the mean age of MRI participants without reported IF in exam 2 was 57.7 ± 5.7 years. 50% (n = 25) of MRI participants with reported IF and 57% (n = 92) of participants without IF were male and the mean BMI was 28.3 ± 4.1 kg/m^2^ and 28.2 ± 4.9 kg/m^2^, respectively. Further demographic characteristics of the study population are provided in Table 1.

### 3.2. Psychosocial and Health Outcomes: PHQ-9, DEEX, SF-12, PSS-10, and Somatization Scale

**Figure 2:**
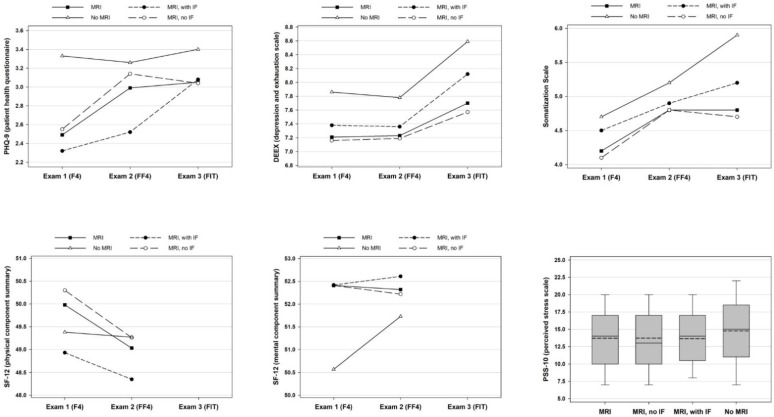
Psychosocial Outcomes of the Study Population according to Participation in Whole-body MRI (MRI) and Presence of Incidental Findings (IF). For PHQ-9, DEEX, SF-12 and the Somatization Scale, arithmetic mean is shown for the four subgroups and three exams. SF-12 was merely applied in exams 1 and 2. Outcomes of PSS-10 obtained in exam 3 are presented as boxplots with arithmetic mean (dashed line) and median (continuous line). Figure 2 shows unadjusted, descriptive data.

Figure 2 shows the trajectories of all psychosocial outcomes in MRI participants, with and without IF, as well as in the control group without MRI participation. The PHQ-9, DEEX and Somatization Scale increased from exam 1 to 3 in all groups. Statistically significant longitudinal changes were observed for PHQ-9 in MRI participants (2.5 to 3.1 points), for DEEX in the control group (7.9 to 8.6 points) and for Somatization Scale in the MRI sample (4.2 to 4.8 points), MRI participants without IF (4.1 to 4.7 points) and the control group (4.7 to 5.9 points). There were no significant differences in psychosocial outcomes at the three timepoints between MRI participants with and without IF.

Non-MRI participants had the highest psychosocial health burden. Compared to MRI participants, they had significantly higher values of PHQ 9 in exam 1 (3.3 ± 3.3 vs. 2.5 ± 2.3) and of DEEX (8.6 ± 4.7 vs. 7.7 ± 4.4), Somatization Scale (5.9 ± 4.3 vs. 4.8 ± 3.8) and PSS-10 (14.7 ± 5.7 vs. 13.7 ± 5.3) in exam 3. Furthermore, the control group had a significantly lower value of MCS obtained by SF-12 than the MRI sample in exam 1, indicating reduced mental health related quality of life (50.6 ± 9.3 vs. 52.4 ± 7.6). MCS and PCS did not differ between groups in exam 2. Further psychosocial outcomes of our study population are shown in Table 2.

### 3.3. Multivariate Analysis of MRI Participation in Association with Psychosocial Outcomes

In adjusted linear mixed models, MRI participation without IF reporting was associated with significantly lower scores of PHQ-9, DEEX and Somatization Scale compared to the control group without MRI participation. The estimated changes of <1 points on the respective scales (Table 3) do not represent clinically actionable differences. MRI participation with IF reporting was also associated with lower scores of all three outcomes; however, the association was not statistically significant nor clinically actionable (Table 3).

Regarding the adjustment variables, there were significant associations of age with higher scores of DEEX and Somatization Scale, of female sex and an intake of antidepressant medication with higher values in all three outcomes, and of BMI and hypertension with higher scores of Somatization Scale. Physical activity was significantly associated with lower values in all three outcomes and living with a partner with lower scores of PHQ-9 and DEEX (Appendix A Table A2).

## 4. Discussion

In this longitudinal, population-based analysis, we investigated long-term associations of MRI participation and IF reporting with psychosocial outcomes in 643 non-MRI participants and 212 MRI participants, of whom 50 had IF.

Non-MRI participants had the highest psychosocial burden. MRI participation without IF reporting was significantly associated with lower values of DEEX, PHQ-9 and Somatization Scale after adjustment for potential confounders. Importantly, there were no significant differences in psychosocial outcomes at the three exams between MRI participants with IF and those without.

We note that men are overrepresented in the MRI group, which is due to the original study design. Since the main aim of the KORA-MRI was to study subclinical disease burden in individuals with prediabetes and diabetes [1], this led to an increased recruitment of men.

Considering the rarity of studies with a comparable design and objective, these results are mainly supported by findings of Schmidt et al., who found no significant adverse long-term psychosocial impact of MRI participation and IF reporting compared to a control group in the Study of Health in Pomerania (SHIP) after 2–3 years of follow-up [38]. In contrast to our study, they assessed intervention effects per year on values of SF-12 and PHQ-9 in a larger sample of 2011 MRI participants and 1735 control subjects in the long-term follow-up survey [38], but did not include data derived prior to MRI. Furthermore, the percentage of disclosed IF was higher in the SHIP cohort (31.5%) [2] than in our MRI sample (22%) [18].

Our mean PHQ-9 values are below the recommended cut-off score of 10 for detection of major depression [32,39] and also lower than reported values of 3.7 ± 3.5 from the German National Cohort [40] and 3.8 ± 3.5 from the SHIP cohort at MRI baseline [38]. The mean values of PCS and MCS we assessed in exam 1 and 2 are also in accordance with the mean PCS and MCS values obtained by Schmidt et al. (48.2 ± 8.1 and 52.8 ± 8.3, respectively) [38] as well as with long-standing values published by Gandek et al. for the German population in 1998 (49.6 ± 8.7 and 52.3 ± 8, respectively) [37]. Furthermore, our outcome values of DEEX are consistent with Ladwig et al., who published a mean DEEX of 8.23 ± 4.76 for the population-based cohort within the Monitoring Trends and Determinants in Cardiovascular Disease (MONICA)/KORA study when they first described the DEEX as a validated screening tool to assess depressive mood in 2004 [29]. The mean PSS-10 values found in our study are slightly elevated for both MRI participants (13.7 ± 5.3) and the control group (14.7 ± 5.7) in comparison to the mean PSS-10 of 11.94 ± 6.14 for individuals aged 60 or older published by Klein et al. based on a representative German community sample [35]. The Somatization Scale is a shortened version of the von Zerssen symptom checklist and has, to our knowledge, not yet been applied in other studies for assessment of somatization symptoms in contrast to a larger, modified version of the von Zerssen symptom checklist, which comprises 24 items [31]. Therefore, the comparability with literature is limited for the Somatization Scale.

Our results showed lower levels of perceived stress, somatization, and depressive mood and exhaustion in our MRI sample compared to the control group in exam 3, i.e., four years after the MRI procedure. This might be explained by a potential reduction of health concerns due to the whole-body MRI scan. This is confirmed not only by our previous finding during the short-term follow-up, in which 88% of MRI participants chose “knowing whether I’m healthy” retrospectively as their main motivation for MRI participation [18], but also by the participants′ common wish for IF reporting in population-based research as a potential expression of interest in their own health and autonomy [18,20]. However, our data showed that MRI participants had less psychosocial burden compared to non-MRI participants already years before the MRI examination. Therefore, our results are in line with the well-known finding that individuals who decide to participate in population-based health examinations are more health-conscious and often healthier than non-participants [41]. We thus cannot conclude that the MRI exam itself represented a beneficial intervention causing improvement in mental health scores. It also needs to be considered that preventive health screenings offered for the general asymptomatic population have been found to be without benefit for participants in terms of total mortality [10] and might cause a high rate of clinically relevant, unexpected findings which require further examinations or surveillance, but frequently turn out to be false positive [7,9].

Nevertheless, our results support the possibility of implementing whole-body MRI in population-based research without overall adverse long-term psychosocial effects even in case of IF disclosure. This finding might have different reasons: First, reported IFs could have mainly turned out to be false positive, although scans had been evaluated by board-certified radiologists [18], due to the high sensitivity of MRI and the low pretest probability of pathological findings in a general population cohort. Second, it is not entirely known if participants followed the recommendations given in IF reports for further examinations as pre- and post-scan survey data were available for only 243 MRI participants in the previously published short-term follow-up [18]. Third, the high quality of our consent form and standardized IF management including IF reports could have positively affected our study results. It is important to provide detailed information in an easily understandable wording to participants to reduce uncertainty in case of IF disclosure and to minimize false expectations regarding the potential disclosure and impact of Ifs, as they might lead to subsequent examinations and could have financial, social and emotional effects [17,19,22,23,24,25]. However, single cases of clinically highly relevant IF with potentially negative long-term psychosocial impacts are not ruled out by our study results as we focused on mean effects. [18]. In this population-based sample, mean values of psychosocial health burden were far below the threshold for clinical disorders. Furthermore, neither IF disclosure nor MRI participation was associated with clinically actionable changes in mental health outcomes.

Our models were adjusted for multiple variables that could possibly confound the association between MRI participation or IF reporting with psychosocial outcomes. In our analysis, female sex was associated with higher values of PHQ-9, DEEX, and somatization. The higher prevalence of depressive and mood disorders in women has been known for a long time [42], which might be due to different etiological factors such as coping mechanisms, response to pharmacological treatment and neurobiology [43]. BMI was associated with increased somatization, but not with depression or exhaustion. Findings on the relation of BMI with mental health burden are inconsistent, but a previous large study from the U.S. showed that unfavorable effects of BMI on depression were only visible in the severe obesity or underweight range [44]. Hypertension was associated with increased somatization. A previous report found that particularly isolated systolic hypertension is associated with somatization [45]. The association between hypertension and depression is still controversial [46,47]. In our sample, smoking was not significantly associated with any mental health parameter. Indeed, previous findings on this association are inconsistent, with both positive and Null findings in prior studies [48]. In the same vein, we did not detect significant associations of diabetes with mental health burden, although such associations have been reported previously [49]. Intake of antidepressant medication was associated with increased mental health burden, which is in line with the interpretation that it served as a proxy to identify affected individuals. Physical activity was associated with decreased mental health burden, which is supported by previous findings [50]. Cohabitation with a partner was associated with decreased mental health burden. This is in line with other studies explaining the protective effect by increased emotional support, intimacy and social network provided by cohabiting partnership [51].

Nevertheless, there are other potential confounders that were not considered in the current analysis. For example, education level, and socioeconomic status on both the individual and community level might influence mental health outcomes [52,53], as well as the willingness to participate in a health study. Future studies should take these factors into account to further characterize the influence of economic status on the association of IF disclosure and psychosocial outcomes.

Limitations of our study include the small sample size with only 50 participants with IF. Further efforts in larger studies, such as the German National Cohort or UK Biobank, are needed as the range of detected IF can vary according to imaging protocol and cohort. Moreover, our data stem from a single-center study, and included only participants without cardiovascular disease and with white ethnicity. Generalizability to other populations, such as high-risk patients, and other ethnicities still needs to be evaluated. Furthermore, not all psychosocial outcomes were available at every exam. Several individuals with missing outcome data had to be excluded, which was due to missing or incomplete assessment of the questionnaires.

Among the strengths of our study is the longitudinal, population-based study design including data before, at, and after the MRI examination. Moreover, we were able to include a control group without MRI participation. Another strength is the application of a panel of complementary scores based on standardized, self-rated questionnaires to assess psychosocial outcomes including depression, exhaustion, perceived stress, and health-related quality of life, which makes our study unique in the context of population-based MRI research.

Our results suggest that whole-body MRI in population-based research is feasible without adverse long-term psychosocial consequences, even in case of IF reporting, and that individuals who voluntarily participate have better mental health even years before MRI. We thus cannot conclude that the MRI examination had a causal beneficial effect on mental health scores. A high quality not only of informed consent procedures and IF reports, but also of a standardized and balanced IF management in imaging research appears to be of rising importance in this context. Standardized reporting of IF might also be beneficial in clinical care, but the transfer of our results to patient cohorts is clearly restricted. Further research regarding long-term psychosocial consequences of MRI participation and IF reporting in larger cohorts and specific subgroups, as well as medical courses after IF disclosures, is needed.

## Figures and Tables

**Table 1:** Demographic Characteristics of the Study Sample according to Participation in Whole-body MRI (MRI) and Presence of Incidental Findings (IF).

	No MRI	MRI	No IF	With IF *	*p*-Value	*p*-Value
	n = 643	n = 212	n = 162	n = 50	(MRI vs. No MRI)	(IF vs. No IF)
Age (years)						
Exam 1 ^a^	51.6 ± 5.6	51.8 ± 5.5	51.4 ± 5.6	53.3 ± 5	0.69	0.03
Exam 2	58 ± 5.5	58.2 ± 5.6	57.7 ± 5.7	59.6 ± 5	0.78	0.04
Exam 3	62.4 ± 5.5	62.4 ± 5.7	61.9 ± 5.8	63.9 ± 5	0.96	0.03
Sex (men)						
Exam 1 ^a^	263 (42%)	113 (56%)	89 (57%)	24 (51%)	0.001	0.58
Exam 2	271 (42%)	117 (55%)	92 (57%)	25 (50%)	0.001	0.5
Exam 3	271 (42%)	117 (55%)	92 (57%)	25 (50%)	0.001	0.5
BMI (kg/m^2^)						
Exam 1 ^b^	27 ± 4.9	27.6 ± 4.5	27.6 ± 4.7	27.6 ± 3.9	0.1	1
Exam 2	27.6 ± 5.3	28.2 ± 4.7	28.2 ± 4.9	28.3 ± 4.1	0.15	0.91
Exam 3	28 ± 5.4	28.5 ± 4.8	28.4 ± 4.9	28.5 ± 4.4	0.24	0.97
Physical activity						
Exam 1 ^a^	408 (65%)	128 (63%)	98 (63%)	30 (64%)	0.6	1
Exam 2	414 (64%)	136 (64%)	105 (65%)	31 (62%)	1	0.85
Exam 3	467 (73%)	140 (66%)	107 (66%)	33 (66%)	0.08	1
Family status (Living together with a partner)						
Exam 1 ^a^	509 (82%)	165 (81%)	127 (81%)	38 (81%)	1	1
Exam 2	488 (76%)	161 (76%)	122 (75%)	39 (78%)	1	0.84
Exam 3	504 (78%)	166 (78%)	126 (78%)	40 (80%)	1	0.89
Diabetes mellitus						
Exam 1 ^a^	31 (5%)	14 (7%)	14 (9%)	0	0.38	0.04
Exam 2	52 (8%)	29 (14%)	25 (15%)	4 (8%)	0.02	0.24
Exam 3 ^c^	45 (7%)	25 (12%)	20 (12%)	5 (10%)	0.04	0.84
Hypertension						
Exam 1 ^d^	163 (26%)	58 (29%)	44 (28%)	14 (30%)	0.56	0.98
Exam 2 ^e^	211 (33%)	84 (40%)	63 (39%)	21 (42%)	0.09	0.82
Exam 3	289 (45%)	110 (52%)	82 (51%)	28 (56%)	0.09	0.61
Intake of antidepressants						
Exam 1 ^a^	26 (4%)	4 (2%)	3 (2%)	1 (2%)	0.2	1
Exam 2 ^e^	34 (5%)	12 (6%)	9 (6%)	3 (6%)	0.98	1
Exam 3 ^e^	35 (6%)	12 (6%)	9 (6%)	3 (6%)	1	1
Smoking status						
Exam 1 ^a^						
Smoker	111 (18%)	47 (23%)	36 (23%)	11 (23%)		
Ex-smoker	268 (43%)	83 (41%)	64 (41%)	19 (40%)		
Never-smoker	245 (39%)	73 (36%)	56 (36%)	17 (36%)	0.24	0.98
Exam 2						
Smoker	99 (15%)	46 (21%)	37 (23%)	9 (18%)		
Ex-smoker	291 (45%)	90 (43%)	67 (41%)	23 (46%)		
Never-smoker	253 (39%)	76 (36%)	58 (36%)	18 (36%)	0.1	0.74
Exam 3						
Smoker	90 (14%)	36 (17%)	28 (17%)	8 (16%)		
Ex-smoker	266 (41%)	91 (43%)	70 (43%)	21 (42%)		
Never-smoker	287 (45%)	85 (40%)	64 (40%)	21 (42%)	0.4	0.95

Table 1 shows unadjusted, descriptive data; *p*-values from repeated measures ANOVA; BMI denotes body mass index; physical activity was defined as self-reported regular physical activity exceeding 1h per week; * denotes at least 1 reported IF; ^a^ denotes n = 624 (No MRI), n = 203 (MRI), n = 156 (No IF), n = 47 (With IF); ^b^ denotes n = 622 (No MRI), n = 203 (MRI), n = 156 (No IF), n = 47 (With IF); ^c^ denotes n = 641 (No MRI); ^d^ denotes n = 623 (No MRI), n = 203 (MRI), n = 156 (No IF), n = 47 (With IF); ^e^ denotes n = 642 (No MRI).

**Table 2:** Characteristics of the Study Sample according to Participation in Whole-body MRI (MRI) and Presence of Incidental Findings (IF) regarding Psychosocial Outcomes. PHQ-9 denotes Patient Health Questionnaire; DEEX, Depression and Exhaustion Scale; Som. Scale, Somatization Scale; SF-12, Short-Form-Health-Survey-12; PCS, physical component summary score; MCS, mental component summary score; PSS-10, Perceived Stress Scale.

	No MRI	MRI	No IF	With IF *	*p*-Value	*p*-Value
	n = 643	n = 212	n = 162	n = 50	(MRI vs. No MRI)	(IF vs. No IF)
PHQ-9						
Exam 1 ^a^	3.3 ± 3.3	2.5 ± 2.3	2.6 ± 2.3	2.3 ± 2.4	<0.001	0.56
Exam 2	3.3 ± 3.1	3 ± 3.2	3.1 ± 3.4	2.5 ± 2.1	0.28	0.23
Exam 3	3.4 ± 3.1	3.1 ± 2.8	3 ± 3	3.1 ± 2.3	0.15	0.94
Longitudinal *p*-value	0.54	0.02	0.06	0.05		
DEEX						
Exam 1 ^a^	7.9 ± 4.7	7.2 ± 4.3	7.2 ± 4.4	7.4 ± 3.9	0.08	0.76
Exam 2	7.8 ± 4.8	7.2 ± 4.6	7.2 ± 4.7	7.4 ± 4.6	0.14	0.82
Exam 3	8.6 ± 4.7	7.7 ± 4.4	7.6 ± 4.5	8.1 ± 4.1	0.01	0.44
Longitudinal *p*-value	<0.001	0.13	0.32	0.32		
Somatization Scale						
Exam 1 ^a^	4.7 ± 4	4.2 ± 3.7	4.1 ± 3.8	4.5 ± 3.6	0.09	0.46
Exam 2	5.2 ± 4.2	4.8 ± 3.7	4.8 ± 3.7	4.9 ± 3.4	0.16	0.86
Exam 3 ^e^	5.9 ± 4.3	4.8 ± 3.8	4.7 ± 3.9	5.2 ± 3.7	0.001	0.4
Longitudinal *p*-value	<0.001	0.013	0.02	0.41		
SF-12						
PCS						
Exam 1 ^b^	49.4 ± 8.2	50 ± 8	50.3 ± 7.6	48.9 ± 9.4	0.37	0.31
Exam 2 ^c^	49.3 ± 8	49 ± 8.4	49.3 ± 8.6	48.4 ± 7.8	0.67	0.2
MCS						
Exam 1 ^b^	50.6 ± 9.3	52.4 ± 7.6	52.4 ± 7.8	52.4 ± 6.9	0.01	1
Exam 2 ^c^	51.7 ± 8.4	52.3 ± 8.5	52.2 ± 8.7	52.6 ± 8	0.14	0.74
PSS-10						
Exam 3 ^d^	14.7 ± 5.7	13.7 ± 5.3	13.7 ± 5.4	13.7 ± 4.7	0.03	0.94

Table 2 shows unadjusted, descriptive data; * denotes at least 1 reported IF; ^a^ denotes n = 624 (No MRI), n = 203 (MRI), n = 156 (No IF), n = 47 (With IF); ^b^ denotes n = 614 (No MRI), n = 201 (MRI), n = 154 (No IF), n = 47 (With IF); ^c^ denotes n = 607 (No MRI), n = 202 (MRI), n = 152 (No IF), n = 50 (With IF); ^d^ denotes n = 621 (No MRI), n = 208 (MRI), n = 159 (No IF), n = 49 (With IF); ^e^ denotes n = 642 (No MRI).

**Table 3:** Association of MRI Participation and IF Reporting with Trajectories of Psychosocial Outcomes. Results from linear mixed regression models with random intercept using data from exams 1–3. β indicates the effect estimate on the outcome for the respective participation category of interest, in units of the outcome. Models were adjusted for age, sex, body mass index (BMI), hypertension (no/yes), smoking status (never, ex, current), diabetes (no/yes), intake of antidepressants (no/yes), physical activity (no/yes) and family status (not living with a partner/living with a partner). MRI denotes magnetic resonance imaging; IF, incidental finding; PHQ-9; Patient Health Questionnaire; DEEX, Depression and Exhaustion Scale; 95%-CI, 95%-confidence interval; ref., reference.

	Outcome PHQ-9			Outcome DEEX			Outcome Somatization		
	β	95%-CI	*p*-Value	β	95%-CI	*p*-Value	β	95%-CI	*p*-Value
Control group (no MRI participation)	ref.	ref.	ref.	ref.	ref.	ref.	ref.	ref.	ref.
MRI participation, no IF	−0.46	[−0.85, −0.04]	0.036	−0.82	[−1.44, −0.14]	0.016	−0.88	[−1.42, −0.31]	0.004
MRI participation, with IF	−0.69	[−1.41, 0.05]	0.064	−0.56	[−1.74, 0.61]	0.362	−0.73	[−1.74, 0.27]	0.172

## Data Availability

Restrictions apply to the availability of these data. Data was obtained from the Helmholtz Zentrum München—German Research Center for Environmental Health and are available from the Helmholtz Zentrum München by submission of a research proposal application.

## Appendix A

**Table A1:** Demographic Characteristics of Excluded Subjects according to Participation in Whole-body MRI (MRI) and Presence of Incidental Findings (IF).

	No MRI	MRI	No IF	With IF *
	n = 2155	n = 188	n = 149	n = 39
Age (years)				
Exam 1	58.7 ± 14.7 (n = 2073)	46.9 ± 11.6 (n = 177)	45.5 ± 11 (n = 139)	52 ± 12.5 (n = 38)
Exam 2	62.9 ± 14.9 (n = 1236)	53.4 ± 11.5 (n = 188)	52 ± 10.9 (n = 149)	58.5 ± 12.4 (n = 39)
Exam 3	62.2 ± 6.3 (n = 74)	65.5 ± 5.7 (n = 6)	63.3 ± 5.3 (n = 4)	70 ± 4.2 (n = 2)
Sex (men)				
Exam 1	998 (48%) (n = 2073)	111 (63%) (n = 177)	89 (64%) (n = 139)	22 (58%) (n = 38)
Exam 2	600 (49%) (n = 1236)	114 (61%) (n = 188)	91 (61%) (n = 149)	23 (59%) (n = 39)
Exam 3	32 (43%) (n = 74)	5 (83%) (n = 6)	3 (75%) (n = 4)	2 (100%) (n = 2)
BMI (kg/m^2^)				
Exam 1	27.8 ± 4.9 (n = 2058)	27.2 ± 4.3 (n = 177)	27.2 ± 4.4 (n = 139)	27.3 ± 4 (n = 38)
Exam 2	27.8 ± 4.9 (n = 1234)	28 ± 5.2 (n = 188)	27.9 ± 5.4 (n = 149)	28.4 ± 4.2 (n = 39)
Exam 3	28.1 ± 5.4 (n = 74)	30.1 ± 3.2 (n = 6)	28.7 ± 2.9 (n = 4)	32.8 ± 1.7 (n = 2)
Physical activity				
Exam 1	1044 (50%) (n = 2068)	91 (51%) (n = 177)	69 (50%) (n = 139)	22 (58%) (n = 38)
Exam 2	653 (53%) (n = 1236)	102 (54%) (n = 188)	79 (53%) (n = 149)	23 (59%) (n = 39)
Exam 3	50 (69%) (n = 73)	1 (17%) (n = 6)	1 (25%) (n = 4)	0 (0%) (n = 2)
Family status (Living together with a partner)				
Exam 1	1499 (72%) (n = 2070)	140 (79%) (n = 177)	109 (78%) (n = 139)	31 (82%) (n = 38)
Exam 2	886 (72%) (n = 1236)	143 (76%) (n = 188)	113 (76%) (n = 149)	30 (77%) (n = 39)
Exam 3	53 (72%) (n = 74)	4 (67%) (n = 6)	2 (50%) (n = 4)	2 (100%) (n = 2)
Diabetes mellitus				
Exam 1	295 (14%) (n = 2073)	11 (6%) (n = 177)	8 (6%) (n = 139)	3 (8%) (n = 38)
Exam 2	222 (18%) (n = 1236)	24 (13%) (n = 188)	17 (11%) (n = 149)	7 (18%) (n = 39)
Exam 3	8 (11%) (n = 73)	0 (0%) (n = 6)	0 (0%) (n = 4)	0 (0%) (n = 2)
Hypertension				
Exam 1	913 (44%) (n = 2065)	41 (23%) (n = 177)	29 (21%) (n = 139)	12 (32%) (n = 38)
Exam 2	545 (44%) (n = 1232)	52 (28%) (n = 188)	38 (26%) (n = 149)	14 (36%) (n = 39)
Exam 3	38 (52%) (n = 73)	1 (17%) (n = 6)	0 (0%) (n = 4)	1 (50%) (n = 2)
Intake of antidepressants				
Exam 1	96 (5%) (n = 2073)	1 (1%) (n = 177)	1 (1%) (n = 139)	0 (0%) (n = 38)
Exam 2	78 (6%) (n = 1233)	5 (3%) (n = 188)	5 (3%) (n = 149)	0 (0%) (n = 39)
Exam 3	5 (7%) (n = 73)	0 (0%) (n = 6)	0 (0%) (n = 4)	0 (0%) (n = 2)
Smoking status				
Exam 1	n = 2067	n = 177	n = 139	n = 38
Smoker	353 (17%)	36 (20%)	30 (22%)	6 (16%)
Ex-smoker	823 (40%)	73 (41%)	53 (38%)	20 (53%)
Never-smoker	891 (43%)	68 (38%)	56 (40%)	12 (32%)
Exam 2	n = 1156	n = 177	n = 139	n = 38
Smoker	150 (13%)	31 (18%)	25 (18%)	6 (16%)
Ex-smoker	490 (42%)	78 (44%)	58 (42%)	20 (53%)
Never-smoker	516 (45%)	68 (38%)	56 (40%)	12 (32%)
Exam 3	n = 72	n = 4	n = 2	n = 2
Smoker	16 (22%)	0 (0%)	0 (0%)	0 (0%)
Ex-smoker	26 (36%)	1 (25%)	1 (50%)	0 (0%)
Never-smoker	30 (42%)	3 (75%)	1 (50%)	2 (100%)

Table A1 shows unadjusted, descriptive data; BMI denotes body mass index; physical activity was defined as self-reported regular physical activity exceeding 1h per week; * denotes at least 1 reported IF.

**Table A2:** Association of MRI Participation and IF Reporting with Trajectories of Psychosocial Outcomes. Results are from linear mixed regression models with random intercept using data from exams 1–3.

	Outcome PHQ-9			Outcome DEEX			Outcome Somatization		
	β	95%-CI	*p*-Value	β	95%-CI	*p*-Value	β	95%-CI	*p*-Value
Control group (no MRI participation)	ref.	ref.	ref.	ref.	ref.	ref.	ref.	ref.	ref.
MRI participation, no IF	−0.46	[−0.85, −0.04]	0.036	−0.82	[−1.44, −0.14]	0.016	−0.88	[−1.42, −0.31]	0.004
MRI participation, with IF	−0.69	[−1.41, 0.05]	0.064	−0.56	[−1.74, 0.61]	0.362	−0.73	[−1.74, 0.27]	0.172
Age, years	0.00	[−0.01, 0.02]	0.812	0.04	[0.01, 0.06]	0.002	0.06	[0.05, 0.08]	<0.001
Female sex	0.94	[0.61, 1.26]	<0.001	1.59	[1.05, 2.11]	<0.001	1.65	[1.18, 2.09]	<0.001
BMI, kg/m^2^	0.02	[−0.01, 0.05]	0.166	0.02	[−0.03, 0.07]	0.534	0.14	[0.09, 0.18]	<0.001
Hypertension	0.06	[−0.2, 0.33]	0.654	0.18	[−0.2, 0.56]	0.33	0.37	[0.05, 0.7]	0.032
Ex-smoker	0.20	[−0.12, 0.53]	0.248	0.06	[−0.44, 0.55]	0.846	0.05	[−0.38, 0.48]	0.836
Current smoker	0.23	[−0.19, 0.66]	0.272	0.18	[−0.48, 0.81]	0.614	0.25	[−0.31, 0.79]	0.384
Diabetes	0.24	[−0.27, 0.72]	0.33	0.10	[−0.66, 0.8]	0.798	0.35	[−0.3, 0.95]	0.266
Intake of antidepressants	1.71	[1.18, 2.23]	<0.001	1.71	[0.99, 2.46]	<0.001	0.76	[0.14, 1.4]	0.022
Physically active	−0.36	[−0.59, −0.12]	0.002	−0.81	[−1.14, −0.47]	<0.001	−0.57	[−0.86, −0.28]	<0.001
Living with partner	−0.85	[−1.18, −0.53]	<0.001	−0.56	[−1.02, −0.1]	0.016	−0.37	[−0.77, 0.02]	0.058
